# Building Numeracy Skills: Associations between DUPLO^®^ Block Construction and Numeracy in Early Childhood

**DOI:** 10.3390/jintelligence11080161

**Published:** 2023-08-10

**Authors:** Katie A. Gilligan-Lee, Elian Fink, Lewis Jerrom, Megan P. Davies, Caoimhe Dempsey, Claire Hughes, Emily K. Farran

**Affiliations:** 1 School of Psychology, University College Dublin, D04 V1W8 Dublin, Ireland; 2 Centre for Educational Neuroscience, Birkbeck, University of London, London WC1H 0AL, UK; 3 School of Psychology, University of Sussex, Falmer, East Sussex BN1 9QH, UK; 4 School of Psychology, University of Surrey, Guildford GU2 7XH, UK; 5 Centre for Family Research, University of Cambridge, Cambridge CB2 3RQ, UK

**Keywords:** numeracy, spatial skills, block construction, early years, socio-economic status

## Abstract

Research shows that children’s block construction skills are positively associated with their concurrent and later mathematics performance. Furthermore, there is evidence that block construction training is particularly beneficial for improving early mathematics skills in children from low-Socio Economic Status (SES) groups who are known to have lower maths performance than their peers. The current study investigates (a) the association between block construction and mathematics in children just before the start of formal schooling (4 years-of-age in the UK) and (b) whether the association between block construction and mathematics differs between children from more compared to less affluent families. Participants in this study included 116 children (M = 3 years 11 months, SD = 3 months) who all completed numeracy, block construction, and receptive vocabulary tasks. Socio-economic status and demographic information (child age, gender, ethnicity) were also obtained from parents. Findings show a strong positive association between block construction and early numeracy skills. Block construction skills explained approximately 5% of the variation in numeracy, even after controlling for age in months, household income, and child receptive vocabulary. When separated by SES group, for children from less affluent families, block construction explained a significant amount of variability (14.5%) in numeracy performance after covariates. For children from more affluent families, block construction did not explain a significant amount of variation in numeracy. These findings suggest that, interventions involving block construction skills may help to reduce SES-based attainment gaps in UK children’s mathematics achievement.

## 1. Introduction

Block construction toys such as LEGO^®^ are extremely popular, and evidence suggests that block construction skills in childhood are positively related to mathematics performance, cross-sectionally (e.g., Nath and Szücs 2014), and longitudinally (e.g., Verdine et al. 2014a). There is also some preliminary evidence that training block construction skills can improve children’s mathematics performance (Newman et al. 2020; although, see Bower et al. 2020a; Schmitt et al. 2018). Therefore, and as outlined by Verdine et al. (2014b), early spatial skills may be key for school readiness in Science, Technology, Engineering, and Mathematics (STEM) subjects. Despite evidence that block construction skills are positively related to mathematics in preschoolers and older children, no studies have specifically investigated this relationship at the age at where children enter formal education. Here, we address this gap by examining block-construction–numeracy relations at the age where children enter formal schooling in the UK, aged 4 years. There are both theoretical and practical implications of extending the previous block construction literature to include this population. First, at four years-of-age, children in the UK have experienced the cumulative effects of nursery, early years childcare, and home environments; however, they have not yet received any standardised curriculum training. This cusp of transitioning to formal education gives insight into the effects of informal early experiences on block construction and numeracy that can be disentangled from any formal educational effects. Second, our study has particular relevance for Reception teachers in the UK who are tasked by the UK government to reduce attainment gaps when children enter school (during their first school year in the UK, children must turn 5 years old). Identifying possible intervention targets for improving attainment in numeracy at this age is therefore pertinent for UK-based educators, particularly given that poverty-led attainment gaps in cognitive performance are already present by 3, 5, and 7 years of age in the UK, including significantly lower mathematics performance in 7-year-olds from socially disadvantaged groups (Dickerson and Popli 2016). Thus, we also investigate whether relations between block construction and mathematics differ across income groups, i.e., between more and less affluent families. Finally, although there are similar studies on children at 3 and 6 years, this study helps to fill in a missing piece in the developmental puzzle on block-construction–numeracy associations through early childhood.

### 1.1. Block Construction and Mathematics

Block play can be broadly sub-divided into two types. In free block play, children can use the blocks provided to build any structure or design they wish. In structured block play, children are given a set of blocks and are required to build a particular structure by copying a target model. Verdine et al. (2014c) proposed that these types of block play recruit different skills, such that free block play calls on creativity and imagination to generate complex relations, while structured block play requires children to analyse a given spatial representation. Expanding on this, Casey and Bobb (2003) proposed that structured block play requires several skills that are important for mathematics including estimation, measurement, patterning, part–whole relations, visualization, symmetry, transformation, and balance. Therefore, the focus of this study, and the remaining literature review, is on structured block play only. Note that guided play is another term used to describe block play scenarios. Some studies use the terms structured and guided play synonymously, referring to block construction where a specific target model is provided as a goal (Ferrara et al. 2011). However, other studies use the term guided block play to describe a scenario where children are given a broad narrative goal for their build, e.g., build a tower, but no target model is specified (Casey et al. 2008; Ramani et al. 2014). Guided block play of this type is also beyond the scope of this study.

#### 1.1.1. Evidence from Preschool Populations

Several studies have investigated the relations between structured block construction and mathematics in preschool children. Verdine et al. (2014c) explored block construction skills in 3-year-olds using the Test of Spatial Assembly (TOSA), a measure very similar to the one used in the current study. Block construction performance was quantified in two ways, using a simple match score (either 100% accurate compared to the target model or not) and a more detailed dimensional scoring system. Block construction match scores explained 15% of the variance in mathematics performance, specifically an assessment measuring number and operations skills, even after controlling for language ability. However, dimension scores explained no additional unique variation. In a follow-up longitudinal study, Verdine et al. (2014a) found that in combination, block construction and visual–motor integration skills at 3 years predicted 27% of the variation in mathematics problem solving at age 4, even after controlling for vocabulary skills and executive functioning (inhibitory control and cognitive flexibility). Bower et al. (2020b) also found that scores related to the structural complexity of children’s block construction on the TOSA, measured by the number of bricks partially overlapping, and perpendicularly placed above underlying bricks, were significantly associated with concurrent mathematical skills (number, operation, and counting skills) in children aged 3 years. In another study by this group, a causal effect of block construction on mathematics was demonstrated in 3-year-olds such that structured spatial construction training was shown to improve mathematics outcomes for children. Interestingly, this improvement was specific to children from low socio-economic-status (SES) backgrounds (Bower et al. 2020a). However, not all studies support a causal effect of block construction on numeracy. Schmitt et al. (2018) completed a block construction intervention with 3–5-year-olds and found no significant effect on numeracy outcomes. However, the analysis was underpowered to find small effects (*N* = 59), and the authors commented on the favourable effect sizes given the restricted power levels.

#### 1.1.2. Evidence from Primary School Populations

Evidence for an association between block construction and mathematics can also be taken from studies of older, primary-school-aged children who have greater statistical power and thus can detect smaller effects. Thomson et al. (2018) found that block construction accuracy was significantly associated with performance on Woodcock–Johnson Applied Mathematics problems in 6- to 7-year-olds (*N* = 104). Similarly, Richardson et al. (2014) found that Lego construction task performance in 7- to 8-year-olds was significantly correlated with mathematics achievement (standard UK Curriculum tests known as SATs) (*N* = 96). Nath and Szücs (2014) replicated and expanded these findings, reporting that visuo-spatial working memory ability in 7-year-olds mediates positive associations between Lego construction and mathematical skills (*N* = 66). More recently, McDougal et al. (2023) reported both direct and indirect (mediated by numerous spatial skills) associations between Lego construction and mathematical skills, explaining up to 26.5% of the variation in mathematics performance (*N* = 358). Beyond correlational findings, recent intervention work with this age group provides evidence that structured block construction may enhance mathematics ability. Newman et al. (2020) found that a structured block play intervention led to significantly faster performance on addition and subtraction calculation problems in 7- to 9-year-olds compared to a free-play block intervention that showed no significant gains (note, however, that this was not reflected in an intervention group x pre-test vs. post-test interaction, possibly due to the small sample size of 43). Furthermore, using MRI, they demonstrated that children in the structured block play intervention group showed increased activation in several brain regions after training while children in the free-play group did not.

To summarise, there is evidence that structured block construction ability is related to mathematical skills, with some support for a causal effect.

#### 1.1.3. Theoretical Rationale

Several theoretical explanations may explain the associations between block construction and numeracy. First, block construction could draw on skills that are crucial to developing numeracy and geometry abilities, such as ordering, counting, and understanding shape dimensions (Hirsch 1996). More specifically, LEGO can require measurement concepts such as counting pips and arranging pips, e.g., a 2 × 4 Lego block (Verdine et al. 2014c). Second, there is evidence that block building offers opportunities for mathematics talks about shapes, sizes, and numbers (Ramani et al. 2014). In turn, mathematical and spatial language have been associated with improved mathematics performance in childhood (Gilligan-Lee et al. 2021; Purpura and Reid 2016). Beyond domain-specific skills, block construction is also likely to recruit a composite of domain-general skills, each individually known to be related to mathematics achievement. These include spatial skills (Gilligan et al. 2019; Frick 2019), working memory (Peng et al. 2015; Nath and Szücs 2014), and executive functions (Hawes et al. 2019b). For example, there is evidence that both spatial and mathematic abilities are dependent on the same brain regions in the intra parietal sulcus (Hawes et al. 2019a). Therefore, engaging in spatial activities, such as block construction, may strengthen the neural circuits that are used in mathematics. Similarly, Hawes and Ansari (2020) propose the spatial modelling account to explain the relationship between space and maths; they suggest that spatial visualisation is used in mathematics, such as geometry and arithmetic, where there is a requirement for visualising and mentally manipulating numbers. Block construction similarly recruits spatial visualisation, including mental rotation and mental transformation, during brick placement, which may strengthen spatial visualisation skills (McDougal et al. 2023). We propose that practice with blocks may lead to improvements in any one of these individual skills or indeed several of them in combination. These improvements may in turn have positive implications for mathematics, and thus associations between block construction and numeracy are expected.

### 1.2. Block Construction and SES

Another aim of this study is to explore differences in block construction skills based on differences in family income. In the current study, we use family income as one possible proxy for SES (and in Appendix A, we investigate the effects using an SES composite score). However, it is noteworthy that across studies, different metrics are used to quantify SES. Therefore, for clarity in this section, we have been explicit in describing how SES was determined in each study. Previous studies have reported that children from lower SES families have poorer spatial skills, including performance on spatial assembly (Verdine et al. 2014c; SES determined using the mother’s education level), mental rotation and spatial scaling tasks (Levine et al. 2005; SES determined using median income split), and wider spatial task batteries, including mental rotation, visual motor integration, block design, map reading, and perspective taking (Johnson et al. 2022; SES determined using household income and free school meal status of child’s school). This relatively lower spatial performance may be explained by reduced access to home numeracy resources, including toys such as building blocks in lower SES households (Clerkin and Gilligan (2018): SES determined using parents’ education and access to home resources). Similarly, Levine et al. (2012) found that children from higher-income families played more frequently with puzzles than children from low-income households, where SES was measured using the parents’ education level and household income. However, other studies have found no significant difference in access to spatial play across SES groups (Jirout and Newcombe 2015: SES determined using parents’ education level and household income). Instead, they have suggested that disparities across SES groups may be due to lower *quality* of spatial play for children from lower SES groups, and not reduced *access*. Using observations, Trawick-Smith et al. (2015) investigated differences in the quality of spatial play (a measure assessing problem solving, sustained interest, autonomy, and creativity among others) when 3- and 4-year-old children interacted with nine different spatial toys. They reported significantly lower quality of play for children from low- compared to middle- and high-income families, where income was measured by family eligibility for free school meals. Similarly, Verdine et al. (2014c) found that parents from lower SES families used fewer spatial words with their 3-year-old children, and this was associated with reduced spatial construction skills. Finally, in their study of 7-year-old girls, Dearing et al. (2012) reported that family income was positively associated with spatial activities in the home, and this interaction was mediated by the level of home learning investment, i.e., parents’ investment of time, energy, and resources. SES was measured using household income. Taken together, this evidence may suggest that there is reduced access to and quality of spatial play in lower-income families, which may in turn lead to fewer opportunities for spatial learning and skill development. Here, we extend these findings on spatial skill/play by assessing differences in block construction skills specifically across children from households with lower and higher incomes.

For associations between block construction and mathematical skills, there is evidence that block construction may be particularly important for lower SES groups. Bower et al. (2020b) reported that early block construction skills at 3 years were significant longitudinal predictors of mathematics at 5 years for children from low SES groups only, where SES was determined using mother’s education level. This group also found that spatial training was particularly effective for 3-year-olds from low SES groups, again measured using mother’s education level (Bower et al. 2020a). Schmitt et al. (2018) similarly found greater benefits of a block play intervention on numeracy for 3- to 5-year-olds from low SES families (determined using the parent’s education level). These arguments are further supported by evidence from the wider spatial literature, where it has been found that spatial skills partially mediate SES-based differences in mathematics performance in children (Johnson et al. 2022; SES determined using household income and free school meal status of child’s school). No known studies have investigated SES-based differences in the role of block construction for UK children as they approach formal education. For this reason, the current study investigates the differences in block construction between more and less affluent families and explores the possible interactions between household income and block construction skills in explaining variance in mathematics performance at 4 years, when children in the UK are on the cusp of starting school.

#### 1.2.1. Theoretical Rationale

There are also theoretical explanations for why associations between block construction and numeracy may differ across SES groups. First, spatial strategies to solving mathematics problems, which may be improved/measured through block construction, may be more beneficial to children from lower SES families. There are many known strategies to solving mathematical and numeracy problems including spatial, verbal, memory, and procedural strategies, among others. It has been hypothesised previously that spatial strategies may be more important when solving novel/challenging activities compared with acquired mathematical skills/activities (Mix et al. 2016). Given that children from lower SES families have been found previously to have poorer mathematics performance than their higher SES peers (e.g., Gilligan et al. 2017: SES determined using household income), they may find mathematics content more challenging. All the while that mathematics content is challenging, they rely on spatial strategies more frequently than other strategies. By extension, children who have mastered a mathematical skill may transition from using spatial strategies to implementing new verbal and memory strategies. Therefore, children from higher-income families may experience a reduced role for spatial skills because they use other strategies. Thus, rendering stronger associations between block construction and numeracy for lower SES groups. An alternative hypothesis may be that spatial and mathematical skills are not linearly related. Instead, children may require a certain minimum level of spatial skill for successful application in mathematics. If there are relatively lower spatial starting points in block construction for low SES children compared to their higher SES peers (as outlined previously), this may act as a limiting factor or barrier that impedes the learning of new mathematics/numeracy material in lower SES children only, and consequently leads to stronger block-construction–numeracy associations for this group.

#### 1.2.2. Block Construction and Gender

Gender is included as a covariate in the current study as there are inconsistent previous findings on gender differences in spatial skills (see Newcombe 2020). There is some evidence that males outperform females in spatial tasks from as young as 3 to 4 months and that these gender differences continue to increase with age (e.g., Linn and Petersen 1985; Levine et al. 1999, 2005). However, other studies have not found any gender differences in spatial performance (e.g., Gilligan et al. 2017). For block construction specifically, many of the previously described studies did not report gender comparisons. For those that do report gender comparisons, neither Verdine et al. (2014c) nor Zhang et al. (2020) found gender differences in 3- or 4-year-olds’ spatial construction skills, respectively. However, a gender difference in construction, favouring males, has been found in older children, e.g., adolescents (Casey et al. 2012).

#### 1.2.3. Current Study

The first aim is to explore the associations between block construction and early numeracy skills in UK children aged 4 years, who are at the cusp of starting education. We hypothesise strong positive correlations between block construction ability and numeracy even after controlling for covariates. The second aim is to investigate whether the associations between block construction and numeracy differ between families with higher and lower incomes. Note that while the household income for our sample is not representative of the UK overall, the wide range of scores we observed on the ladder of social standing suggests that the sample represent a sizeable range of economic access to resources. We predict that children from less affluent families will have lower block construction scores but that relations between block construction skill and numeracy outcomes will be strongest in this group. These findings may highlight the potential of a specific advantage of spatial intervention for children from less affluent families, which may in turn offer a novel approach for reducing early attainment gaps in mathematics, which is an educational priority in the first year of schooling in the UK.

## 2. Materials and Methods

### 2.1. Participants

This study recruited 118 first born (eldest) children and their parents. However, only participants with a full set of numeracy data, our primary dependent variable, were included. Thus, the final sample size is 116 participants (42% female), between the ages of 3 years 5 months and 4 years 7 months (3 years 11 months ± 3 months; mean ± SD). All children were tested prior to entering formal education, i.e., before starting their Reception year. Participants were recruited from a larger pool of families who participated in the New Father and Mothers study (Hughes et al. 2018), a study of first-time parents and their children from infancy to 24 months. Families were excluded if either parent had history of severe mental illness or substance abuse. None of the children were bilingual, and all children spoke English in the home. The sample was further divided into lower-income (less affluent, *n* = 60) and higher-income (more affluent) groups (*n* = 56) using a median split of overall household income. The groups are slightly uneven, as 5 participants had the median score (GBP 4500 median household income per month) and were assigned to the lower-income group. Descriptive information on the two groups including gender and other measures of SES can be found in Table 1.

### 2.2. Procedure

This is an associational study that includes data collected from both children and their parents. The study was conducted according to the guidelines of the Declaration of Helsinki and approved by the Ethics Committee of the University of Cambridge. Informed consent was obtained from all subjects involved in the study. Although each child–parent dyad completed a larger battery of tasks, only tasks relevant to the current study will be outlined here. Unless otherwise stated below, tasks were completed in a single session, with the order of these tasks counterbalanced and videotaped for later coding. Parents completed questionnaires while their child was completing the individual tasks with a trained graduate researcher. At the conclusion of the session, families received GBP 10 as compensation for travel costs.

### 2.3. Measures

#### 2.3.1. Block Construction Task

**Figure 1:**
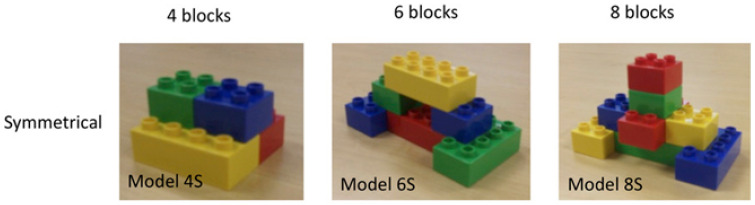
Block models (Figure adapted with permission from Shelton et al. (2022) DOI: 10.1111/cogs.13081).

Children’s block construction ability was assessed by asking children to copy Duplo target models. Duplo is a construction toy like Lego but that uses larger bricks that are more suitable for younger children. The task included four trials, one practice and three experimental (containing different target models). Practice and target models were a subset of those used by Landau et al. (2022) and Shelton et al. (2022). The procedure for this task was also similar to the TOSA; however, the task was coded differently (more details below). For each model, children were given the exact bricks required and were instructed to copy the experimenter’s model exactly. The practice trial, of two blocks, was repeated until participants made a successful replica of the target model. No child required more than two attempts to accurately complete the practice trial. After the practice trial, children progressed to the experimental models. These included 4, 6, and 8 blocks of different colours (red, green, blue, and yellow) and sizes (square 2 × 2 blocks and rectangular 2 × 4 blocks), respectively (Figure 1). For experimental trials, if the child’s model was not a perfect replica, the child was instructed to try again (up to a maximum of three attempts), before moving onto the next trial. However, only the first attempt was scored. For both practice and experimental trials, verbal feedback was given outlining whether the child’s model was correct or incorrect. No other explanation or help was given to assist the child in completing the models. The coding strategy was based on McDougal et al. (2023). There were three coding elements, which were summed together to create a single block construction score (max score: 35). The score was designed to be a sensitive measure of accuracy in both the 2-dimensional structure of each row (row score), the 3-dimensional relationship between the rows (pip placement score), and representation of the whole structure as an integrated model (integration score). Our models were more sophisticated than the TOSA models (TOSA models used 2 to 4 bricks per model), and so the TOSA coding elements would not have been suitable here. However, our coding elements incorporate the principles used to code TOSA models. TOSA ‘vertical location’ is accounted for via our ‘row’ score; TOSA ‘translation’ and TOSA ‘rotation’ are accounted for by our ‘pip placement’ and ‘integration’ scores. The exact coding method is included in Appendix A.

#### 2.3.2. Preschool Early Numeracy Skills Screener–Brief (PEN-B)

Children were assessed on their mathematics ability using the PENS-B (Purpura et al. 2015). This measure contains 24 items on the key domains required for the development of early numeracy skills, including one-to-one counting (e.g., child counts a set of dots), numerical identification (e.g., child has to identify the number presented on a series of flashcards), story problems (e.g., child presented with a story problem containing basic sums such as 4-1), and ordinality (e.g., child has to identify the eighth object). Items are ordered in terms of difficulty, starting from easiest, and the test takes approximately 5 min to administer. For 21 of the items, researchers presented visual stimuli and for the other 3, they read a story. The child was then required to either point to the correct answer or to respond verbally. A point was given per correct answer, and testing was stopped if a child provided three consecutive incorrect responses. A sum of points was then calculated for each child, and a percentage accuracy was generated.

#### 2.3.3. Receptive Vocabulary

Children completed the receptive vocabulary sub-test of the Wechsler Preschool and Primary Scale of Intelligence (WPPSI; Wechsler 2012), a 38-item measure of receptive language skills with excellent test–retest reliability and validity. Children were required to select the picture that best represented the word said aloud by the researcher (out of four possible options). If a child made five consecutive errors, the tasks was ended. Correct answers were summed to create a final receptive vocabulary score out of 38, which was then converted to a percentage accuracy score. To allow age to be independently examined in the models, receptive vocabulary scores were not age-adjusted.

#### 2.3.4. Parental Measures

The primary indicator of social economic status (SES) used in this study was household income, i.e., parents’ combined average monthly income in pounds, which was collected using a self-report questionnaire. Other secondary indicators of SES are reported to provide a more detailed profile of the families in our more and less affluent groups. These measures are legacy data collected at different timepoints from the rest of the data included in this study. First, parents completed the Subjective Social Status Ladder (Singh-Manoux et al. 2003) 4 months after the birth of their child. This scale assesses perceived socio-economic status and requires participants to rank their social standing on a 10-rung ladder. Second, parents reported on the highest level of education they had earned, and participants (families) were allocated to one of two groups based on whether at least one parent in the family had a higher degree (MSC/Doctorate) or not. These data were collected from families prenatally. We also created a composite score for SES through factor analysis with our three SES variables: monthly household income, mean scores on the ladder of social standing per family, and highest level of education.

### 2.4. Data Analysis

Within the Duplo task, some participants were missing data for a single trial (*n* = 8). One participant had missing data for model 1 (4-block model) only. They achieved full marks on both 6- and 8-block models and were thus awarded full marks for model 1. For participants missing data for a single model that was model 2 or model 3 (*n* = 7), their missing score was replaced with the same percentage accuracy as their performance on their complete model, e.g., if a participant was missing data for model 2, their missing score was replaced with their percentage accuracy for model 3. All other missing data (Duplo *n* = 13; SES *n* = 3) was replaced using multiple imputation (MI). Based on Graham et al.’s (2007) guidelines for MI, with 11% missing data (for the variable with the highest fraction of missing data) and a 1% tolerance for statistical power falloff, 20 imputations were completed. All variables with significant correlations to the variables of interest were included as auxiliary variables in MI. Note that when the analyses were completed with complete case data only, the same pattern of results was observed.

Analyses were completed using SPSS. Power analysis for the largest analysis included in this study (a regression analysis with 5 predictors, a medium-sized effect (f = 0.15), power of 80%, and an alpha value of 0.05) revealed that 92 participants were required. This sample size was achieved. Gender differences in child variables were investigated using independent *t*-tests. Where significant, gender was included as a predictor in subsequent regression models. A correlation matrix was used to investigate the relative associations between continuous measures and to inform subsequent general linear models. Regression models were used to explore the contribution of Duplo scores to numeracy performance. Adjusted R^2^ values are reported throughout, and all continuous variables were z-scored prior to regression. A full sample stepwise regression was completed with numeracy scores as the outcome variable. Covariates of age in months, household income, and child receptive vocabulary were entered in Step 1. Duplo scores were entered in Step 2. The interaction between Duplo scores and household income was entered in Step 3. Two methods were used to explore the interaction between Duplo scores and household income. First, simple slopes analysis was completed by investigating associations between block construction and numeracy at high and low values of household income, respectively, i.e., +/− one standard deviation from the income mean. Second, follow-up regression models were completed with the lower-income and higher-income groups separately. To maximise statistical power, only significant predictors from the original model (full sample) were included. Power analysis indicated that for a regression model with a medium-sized effect (f = 0.15), power of 80%, an alpha value of 0.05, and 3 predictors, a minimum of 55 participants is required. This sample size was met for both income groups. We also repeated our main analyses using our composite SES score in place of household income. The results can be found in Appendix A.

## 3. Results

Table 1 shows the descriptive statistics for each variable, and Table 2 shows the descriptives spilt by income group. There were some violations of assumptions of normality; however, in accordance with the Central Limit Theorem (Field 2018), given that our sample size was higher than 30, parametric statistics are used throughout. Children’s numeracy scores were significantly correlated with the other child variables, thus supporting their inclusion in subsequent regression models. Duplo performance was also significantly correlated with income scores. Pearson correlations between all variables are shown in Table 3. T-tests indicated no significant gender differences for any of the child variables, including numeracy, *t*(114) = 1.51, *p* = .130, *d* = 0.285 (males 45.9 ± 2.1; females 50.7 ± 2.3); Duplo, *t*(114) = 0.96, *p* = .336, d = 0.184 (males 68.8 ± 3.8; females 64.3 ± 4.2); or receptive vocabulary scores, *t*(114) = 1.80, *p* = .073, d = 0.337 (males 63.6 ± 1.5; females 67.6 ± 1.6), and all of the effects are small (<0.20) to medium (<0.50) in size (Cohen 1988). Due to the absence of any significant gender effects, gender was not included as a control in the subsequent regression analysis.

In the regression model 1, we explored whether the Duplo scores explain additional variation in numeracy after controlling for covariates. Across all models, all variables entered were continuous. The control variables, receptive vocabulary, household income, and age entered in Step 1 explained 26.8% of the variation, *F* (3, 112) = 15.06, *p* < .001. Duplo scores were entered in Step 2, explaining an additional 5% of the variation in numeracy, *F* (4, 111) = 14.49, *p* < .001. An interaction term between Duplo scores and household income was entered in Step 3, and it explained an additional 2.1% variation, *F* (5, 110) = 12.81, *p* < .001. Duplo scores, receptive vocabulary, and the interaction term were significant in the final model (see Table 4). Based on the beta values and the additional variation explained by the interaction term, the interaction between Duplo scores and household income was further investigated in two ways. First, a simple slope analysis was used to compare the expected values of numeracy skill at high and low values of income. There was a significant difference between the beta values for block construction at high (β = −0.028) and low values (β = 0.399) of income, i.e., +/− one standard deviation from the income mean, *t* (198) = 24.77, *p* < .001, *d* = 3.07. This shows that the association between block construction and numeracy is significantly stronger for low-income families.

Second, we completed two follow-up regressions with lower- and higher-income groups, respectively. Model 2 was completed with participants in the lower-income group only. The control variables entered in Step 1 explained 21.1% of the variation, *F* (2, 57) = 8.88, *p* < .001. Duplo scores were entered in Step 2, explaining an additional 14.5% of the variation in numeracy, *F* (3, 56) = 11.93, *p* < .001. Receptive vocabulary and Duplo scores were significant predictors in the final model (see Table 4). Model 3 was completed with participants in the higher-income group only. The control variables that were entered in Step 1 explained 31.4% of the variation, *F* (2, 53) = 13.58, *p* < .001. Duplo scores were entered in Step 2 and explained no additional variation in numeracy (adjusted *R*^2^ = 0.305). Receptive vocabulary was the only significant predictor in the final model (see Table 4). To ensure that the results for the high-income group could not be explained by ceiling effects, a one-way t-test comparing performance on block construction to ceiling performance (100%) found that the high-income group was not at the ceiling, *t*(55) = 7.317, *p* < .001, *d* = 0.031.

For all the regression models, the collinearity statistics fell within acceptable levels, i.e., VIF (cut-off < 10) (Myers 1990) and tolerance (cut-off > 0.2) (Menard 1995). Furthermore, we repeated all analyses using a composite SES score in place of household income. The results can be found in Appendix A. It is noteworthy that for the SES composite, the interaction term between Duplo scores and SES was not significant (*p* = 0.197); however, the beta value (0.13) was comparable to the model presented in the main paper (0.17). For consistency with the main paper, follow-up regression for high- and low-income groups and simple slope analyses were completed. The patterns of results from these analyses using an SES composite mirrored those in the main paper using household income.

## 4. Discussion

The findings show a strong positive association between block construction and early numeracy skills at the age where children enter formal education in the UK. Block construction skills explained approximately 5% of the variation in numeracy, even after controlling for age in months, household income, and child receptive vocabulary. Receptive vocabulary is used as a proxy for IQ in this study, as also seen in Hodgkiss et al. (2018) and Gilligan et al. (2019). These findings complement and extend previous research such that structured block play has been associated with mathematics performance at various ages, including preschoolers (age 3) (see Verdine et al. 2014a, 2014c; Bower et al. 2020b) and older, primary school children (ages 6–8; see Thomson et al. 2018; Richardson et al. 2014; Nath and Szücs 2014). This study extends the evidence on the associations between block construction and mathematics by investigating a population of children before they first enter formal education in the UK. Given the age group included, the findings provide measures of block construction and numeracy performance from children that have not yet been influenced by formal educational learning, i.e., the experiences influencing the development of these skills in our sample are due to the cumulative effects of nursery, early years childcare, and home environments. This means, for example, that associations between the skills are unlikely to be explained by children learning spatial problem-solving strategies for numeracy. Furthermore, these findings highlight one possible intervention target for promoting maths-readiness in children of this age. Studying this age group of children has particular relevance for Reception teachers in the UK, who are tasked by the UK government to reduce attainment gaps when children enter school (at 4 years).

As outlined in the Introduction, several theoretical explanations may explain the observed associations between block construction and numeracy. Block construction could draw on skills that are crucial to developing numeracy and geometry abilities, such as ordering, counting, and understanding shape dimensions (Hirsch 1996). Block building may offer opportunities for mathematics talks about shapes, sizes, and numbers (Ramani et al. 2014). Finally, block construction may improve other domain-general skills that are important for mathematics, including spatial skills (Gilligan et al. 2019; Frick 2019), working memory (Peng et al. 2015), and executive functions (Hawes et al. 2019b). Practice with blocks may therefore lead to improvements in any one of these individual skills or indeed several of them in combination. These improvements may, in turn, have positive implications for mathematics. While these mechanisms cannot be tested in the current associational study, support is offered by McDougal et al. (2023), who determined that the association between Lego construction and maths in 7- to 9-year-olds is mediated by visualisation (measured using mental rotation), visuo-spatial working memory, disembedding (a measure of part–whole processing), and spatial-numerical relationships (measured using a number line task). These findings, along with the suggestions above, provide clear theoretical explanations for the relations between block construction and early mathematics reported here.

We also found that block construction performance differs across household income groups. Children from families with lower incomes had lower block construction performance. This contrast is intriguing and extends the findings reported from low-income samples (e.g., Bower et al. 2020b), given the relative affluence of the families in the current study. Specifically, in the year these families were seen (2019), their mean household monthly income was GBP 4855, as compared with a UK mean of GBP 3033 (Croal 2022). Thus, we might expect even greater income effects if comparisons were made that included the lowest SES families in the UK. Previous work suggests two possible explanations for income-based differences in early block construction. The first of these concerns is limited access to home numeracy/spatial resources, including blocks (Clerkin and Gilligan 2018), which reduces opportunities for spatial learning. Second, Trawick-Smith et al. (2015) have proposed that the quality of spatial play with parents may also be poorer for children in lower SES households, including lower use of parental spatial language during play (see Verdine et al. 2014c). Consistent with this view, children in low-income households have been reported to show higher rates of screen media time, which reduces the time available for hands-on play with spatial toys (Certain and Kahn 2002; Dennison et al. 2002; Duch et al. 2013). Likewise, as noted in the Introduction, empirical studies have shown that it is the quality of spatial play rather than access to spatial resources that distinguishes lower and higher SES groups (e.g., Jirout and Newcombe 2015; Levine et al. 2012).

For our sample, it is worth noting that the more affluent group was more highly educated (46.7% vs. 75.0% of parents had a higher university degree), which may have led to contrasts in the content and quality of parent–child interactions, even within this very educated and affluent overall sample. Equally, however, the income-related contrasts in block construction observed in this study may reflect a third factor. One candidate is differences in parents’ scientific backgrounds, e.g., education and employment; note that there is a significant pay gap between scientific and non-scientific careers (De Vries 2014). Individuals with STEM expertise are known to have stronger spatial skills (Wai et al. 2009), which may lead them to engage more readily and effectively in spatial activities with their children. A second candidate is maternal involvement in paid employment and hence differences in children’s nursery attendance in the early years, which has been reported to have a beneficial effect on children’s cognitive development, including numeracy skills (Sylva et al. 2004). Although there is no research evidence to date, early nursery attendance may also have benefits for block construction.

Relations between block construction skill and numeracy outcomes were also stronger for children from the lower-income group. Block construction explained a greater proportion of the variation in numeracy for children from less affluent families compared to more affluent families. As previously mentioned, a study by Bower et al. (2020b) also found structured block play to be a predictor of later mathematics abilities in preschoolers, but only for children from low-income families. Spatial training studies have also demonstrated that spatial training is more effective and leads to greater gains in numeracy for young children from low-income compared to high-income families (Bower et al. 2020a; Schmitt et al. 2018). One explanation is that spatial strategies may be more important for novel compared with acquired mathematics (Mix et al. 2016). Children who have mastered a mathematical skill may transition from using spatial strategies to implementing new verbal and memory strategies. For our sample, children from higher-income families may have improved mathematical skills, leading to a reduced role for spatial skills as they use other strategies. As outlined above, this may be attributable to familial strengths (both genetic and environmental) in STEM or to differences in nursery attendance across income groups. We hypothesise that differences in early childhood experiences may lead to more formal number learning opportunities and improved numeracy for our higher-income group, thus explaining differences in the association between block construction and numeracy across groups. We tested this anecdotally by running slope functions between block construction and numeracy and found that both linear and quadratic functions were significant fits to the data (*p* < .001). This may suggest that the link between block construction and numeracy is non-linear, i.e., stronger for those with low numeracy ability.

Our findings provide the required evidence to explore whether there is a specific advantage of block construction intervention for children from lower-income families at the age of school entry. Indeed, given the trends we have reported, we would expect that a lower-income group (lower than the less affluent group in this study) would show even more spatial disparity and greater spatial-mathematics relations than the current sample (e.g., Bower et al. 2020a). Block construction may offer a novel approach for reducing early attainment gaps in mathematics. Reducing gaps at school entry may be particularly important as there is evidence that attainment gaps at this age have a long-lasting influence on children’s later education outcomes, with children from disadvantaged backgrounds showing minimal changes in achievement gradients between age 5 and 18 years (Barnett and Lamy 2013). Given that individuals already behind their peers at the start of school are more likely to continue performing at a lower ability in higher education, reducing SES-based achievement gaps at entry to formal schooling is vital. More specifically, within a UK context, it is a government aim to reduce attainment gaps in the Early Years Foundation Stage (EYFS). Reception is the only formal school year that is part of the EYFS, and thus our findings may offer a novel perspective for teachers who are working towards achieving this aim.

### Strengths and Limitations

The study is strengthened by the fact that the analyses are adequately powered. As outlined in the Introduction, low power is a common theme in previous research on block construction in preschool children. Our findings provide rigorous, suitably powered evidence of income-based differences in block-construction–numeracy associations on which future interventions can be based. However, it is noteworthy that the results of this study differ slightly depending on the measure used to quantify SES, i.e., income differences as reported in the main paper and the SES composite reported in Appendix A. While the overall trends are similar, this suggests that SES differences should be interpreted cautiously and in the context of the SES metric used. Second, causal explanations must be treated cautiously due to the associational design of this study. The correlations do not explain *why* the relations between block construction and mathematics differ for children from families with high vs. low household incomes. Future intervention research measuring the effects of block construction training is required to determine the direction of causality. Third, our sample may not be generalisable as all participants were recruited from (anonymised for review), which is an affluent area relative to other locations in the UK.

## 5. Conclusions

We demonstrated a strong positive association between block construction and numeracy scores in 4-year-old children, a previously un-researched sub-group of children who are at the age of entering formal education in the UK. We also found that children from lower-income households had lower block construction skills. Despite this, block construction performance was a stronger predictor of numeracy for children from lower-income families. These findings highlight block construction as a possible intervention target to promote mathematics readiness in children prior to starting school. Focusing on the quality of play over quantity, these interventions might include features such as (a) targeted model building using a template/design; (b) building that increases in difficulty level/challenge across sessions; (c) encouraged use of spatial visualization and trial and error during construction; (d) verbal feedback using spatial language and, where required, demonstrative feedback (using embodied action and gesture) from a parent/caregiver/teacher. In particular, block construction interventions may be advantageous in reducing household income-based differences in young children’s overall mathematics achievement, both at entry to education and beyond.

## Figures and Tables

**Table 1:** Descriptive statistics for the full sample.

	N	%	Min	Max	Mean	SD
(A) Child age (years)	116		3.42	4.58	3.95	0.24
(B) Numeracy score (%)	116		4.17	91.67	47.92	16.91
(C) Receptive vocabulary (%)	116		15.79	89.47	65.27	11.88
(D) Duplo score (%)	103		2.86	100.00	66.45	28.58
(E) Household income monthly (GBP)	113		1706.00	11,800.00	4855.28	1848.23
(F) Child gender						
Male	67	57.8				
Female	49	42.2				
(G) Higher university degree (MSC/PhD) ^a^						
Yes	70	60.3				
No	46	39.7				
(H) Mean ladder scores	113		4.5	10.00	7.40	1.04
Five	4	3.4				
Six	11	9.5				
Seven	28	24.1				
Eight	47	40.5				
Nine	18	15.5				
Ten	5	4.3				

^a^ This variable outlines the percentage of families in which at least one parent has a higher degree including an MSc or PhD.

**Table 2:** Descriptive statistics presented for the more and less affluent groups separately.

	Less Affluent Group			More Affluent Group		
	N	Mean	SD	N	Mean	SD
(A) Child age (years)	60	3.94	.23	56	3.97	0.25
(B) Numeracy score (%)	60	45.42	17.27	56	50.60	16.23
(C) Receptive vocabulary (%)	60	64.74	12.86	56	65.84	10.83
(D) Duplo score (%)	55	57.62	30.13	48	76.56	23.11
(E) Household income monthly (GBP)	60	3492.79	736.21	53	6397.73	1475.0
	N	%		N	%	
(F) Child gender						
Male	35	58.3		32	57.1	
Female	25	41.7		24	42.9	
(G) Higher university degree (MSc/PhD) ^a^						
Yes	28	46.7		42	75.0	
No	32	53.3		14	25.0	
(H) Ladder scores						
Five	4	6.7		0	0	
Six	6	10.0		5	8.9	
Seven	19	31.7		9	16.1	
Eight	22	36.7		25	44.6	
Nine	7	11.7		11	19.6	
Ten	2	3.3		3	5.4	

^a^ This variable outlines the percentage of families in which at least one parent has a higher degree, including an MSC or PhD.

**Table 3:** Correlations between all continuous measures included in the study.

	Numeracy	Vocabulary	Duplo	Household Income Monthly	SES Composite
Child age (years)	0.099	0.169	0.026	0.107	0.059
Numeracy score (%)		0.526 ***	0.347 ***	0.152	0.085
Receptive vocabulary (%)			0.170	0.085	−0.021
Duplo score (%)				0.303 **	0.263 **
Household income monthly (GBP)					0.795 ***

*** *p* < .001, ** *p* < .01,

**Table 4:** Regression models showing factors predicting numeracy performance in the full sample (*N* = 116), less affluent group (*n* = 60), and more affluent group (*n* = 56).

	Beta	SE	t	*p*
Model 1 (full sample)				
Step 1				
Age	0.00	0.08	−0.06	0.915
Household income monthly (cont.)	0.08	0.08	1.01	0.339
Receptive vocabulary	0.48	0.08	6.07	<.001
Step 2				
Duplo	0.21	0.08	2.54	0.021
Step 3				
Duplo X income	−0.17	0.08	−2.06	0.05
Model 2 (lower-income group only)				
Step 1				
Household income monthly (cont.)	−0.06	0.29	-0.55	0.585
Receptive vocabulary	0.42	0.10	3.84	<.001
Step 2				
Duplo	0.40	0.10	3.73	<.001
Model 3 (higher-income group only)				
Step 1				
Household income monthly (cont.)	0.03	0.12	0.29	0.771
Receptive vocabulary	0.58	0.12	5.09	<.001
Step 2				
Duplo	0.01	0.13	0.06	0.709

Note. Cont = Entered as a continuous variable.

## Data Availability

The data presented in this study are available on request from the corresponding author.

## Appendix A

### Appendix A.1. Spatial Blocks Coding Method

Row Score (R): 1 point per brick (as used in McDougal et al. 2023)

Each row is coded independently from all other rows in the construction, i.e., independent of the orientation of the row to the rest of the model. To achieve a score of 1, a brick must be accurate (in colour, orientation, and location) relative to other bricks within the same row. Note that if there is just one accurately chosen brick in the row, a score of 1 for the row will be given.

Pip Placement (PP): bottom row not coded, all other rows 1 point per brick (as used in McDougal et al. 2023).

PP refers to the accuracy (colour, orientation, location) of each brick relative to the row below it and is thus, scored from the second lowest row upwards (noting that the PP score is impacted by the accuracy of the row below). Additional bricks or additional rows (relative to the model image) are not penalised because points will be lost by not including the brick(s) in the correct row.

Integration scoring (I): bottom row not coded, all other rows 1 point per brick.

Integration scoring captures whether a child has successfully used a brick such that it connects/ties together bricks in the row below. This score has been included so that colour errors are not overly penalised. This is because a child can lose PP scores if the row below is correct but the colours are oriented the wrong way around. In this case, the model would be fully integrated. Integration scores differentiate this example from models that do not resemble the model image (i.e., they lack accurate integration).

### Appendix A.2. Regression Analyses Completed with SES Composite Score

Note that all analyses described below use the SES composite score as the measure of SES. The results reported in the main manuscript use household income.

In Regression Model 1, we explored whether Duplo scores explain additional variation in numeracy after controlling for covariates. Across all models, all variables entered were continuous. The control variables, receptive vocabulary, SES composite, and age, entered in Step 1, explained 26.7% of the variation, *F* (3, 112) = 14.97, *p* < .001. Duplo scores were entered in Step 2, explaining an additional 4.7% of the variation in numeracy, *F* (4, 111) = 14.18, *p* < .001. An interaction term between Duplo scores and the SES composite was entered in Step 3 and explained an additional 1.2% variation, *F* (5, 110) = 12.18, *p* < .001. Duplo scores and receptive vocabulary were significant in the final model (see Table A1). Although the interaction term was not significant, based on the Beta values, the additional variation explained by the interaction term, and for consistency with the main paper, the interaction between Duplo scores and the SES composite was further investigated in two ways. First, simple slope analysis was used to compare the expected values of numeracy skill at high and low values of SES. There was a significant difference between the Beta values for block construction at high (β = 0.045) and low values (β = 0.353) of SES, *t* (204) = 18.32, *p* < .001, *d* = 2.56. This shows that the association between block construction and numeracy is significantly stronger for lower-SES families.

Second, we completed two follow-up regressions with lower and higher SES groups, respectively. Model 2 was completed with participants in the lower SES group only. The control variables entered in Step 1 explained 22.1% of the variation, *F* (2, 57) = 9.35, *p* < .001. Duplo scores were entered in Step 2, explaining an additional 12.8% of the variation in numeracy, *F* (3, 56) = 11.65, *p* < .001. Receptive vocabulary and Duplo scores were significant predictors in the final model (see Table A1). Model 3 was completed with participants in the higher SES group only. The control variables that were entered in Step 1 explained 31.3% of the variation, *F* (2, 53) = 13.55, *p* < .001. Duplo scores were entered in Step 2 and explained less than 1% of the variance (0.8%); *F* (3, 52) = 9.06, *p* < .001. Receptive vocabulary was the only significant predictor in the final model (see Table A1). For all regression models, the collinearity statistics fell within acceptable levels, i.e., VIF (cut-off < 10) (Myers 1990) and tolerance (cut-off > 0.2) (Menard 1995).

**Table A1:** Regression models showing factors predicting numeracy performance in the full sample (*N* = 116), lower SES group (*n* = 60), and higher SES group (*n* = 56) using the SES composite.

	Beta	SE	t	*p*
Model 1 (full sample)				
Step 1				
Age	−0.01	0.08	−0.12	0.909
SES composite	0.06	0.08	0.72	0.474
Receptive vocabulary	0.50	0.08	6.16	<.001
Step 2				
Duplo	0.22	0.09	2.50	0.013
Step 3				
Duplo X SES composite	−0.13	0.10	−1.30	0.197
Model 2 (lower-income group only)				
Step 1				
SES composite	0.07	0.17	0.42	0.675
Receptive vocabulary	0.39	0.10	3.83	<.001
Step 2				
Duplo	0.36	0.11	3.22	<.001
Model 3 (higher-income group only)				
Step 1				
SES composite	−0.04	0.12	−0.30	0.764
Receptive vocabulary	0.61	0.12	5.03	<.001
Step 2				
Duplo	−0.01	0.15	0.07	0.948
